# Krüppel-like Factor-9 and Krüppel-like Factor-13: Highly Related, Multi-Functional, Transcriptional Repressors and Activators of Oncogenesis

**DOI:** 10.3390/cancers15235667

**Published:** 2023-11-30

**Authors:** Frank A. Simmen, Iad Alhallak, Rosalia C. M. Simmen

**Affiliations:** 1 Department of Physiology & Cell Biology, University of Arkansas for Medical Sciences, Little Rock, AR 72205, USA; ialhallak@uams.edu (I.A.); simmenrosalia@uams.edu (R.C.M.S.); 2 The Winthrop P. Rockefeller Cancer Institute, University of Arkansas for Medical Sciences, Little Rock, AR 72205, USA

**Keywords:** Krüppel-like factor 9 (KLF9), Krüppel-like factor 13 (KLF13), oncogenesis, miR, lncRNA, pathways, future directions

## Abstract

**Simple Summary:**

Krüppel-like Factor-9 (KLF9) and Krüppel-like Factor-13 (KLF13) are highly related proteins that function as inducers and/or repressors of specific target gene repertoires in a variety of tissues and in diverse pathophysiological states, including neoplasia. Here, we describe the salient features of KLF9 and KLF13, the current state-of-the-art research regarding both protein’s actions in cancer development and response to therapies, and where the field requires further exploration. These paralogous proteins warrant further study for multiple cancers, and with respect to their multiplicities of action in suppression or promotion of proliferative and metastatic phenotypes, and likely involvement in immune cell biology within the tumor microenvironment.

**Abstract:**

Specificity Proteins/Krüppel-like Factors (SP/KLF family) are a conserved family of transcriptional regulators. These proteins share three highly conserved, contiguous zinc fingers in their carboxy-terminus, requisite for binding to cis elements in DNA. Each SP/KLF protein has unique primary sequence within its amino-terminal and carboxy-terminal regions, and it is these regions which interact with co-activators, co-repressors, and chromatin-modifying proteins to support the transcriptional activation and repression of target genes. Krüppel-like Factor 9 (KLF9) and Krüppel-like Factor 13 (KLF13) are two of the smallest members of the SP/KLF family, are paralogous, emerged early in metazoan evolution, and are highly conserved. Paradoxically, while most similar in primary sequence, KLF9 and KLF13 display many distinct roles in target cells. In this article, we summarize the work that has identified the roles of KLF9 (and to a lesser degree KLF13) in tumor suppression or promotion via unique effects on differentiation, pro- and anti-inflammatory pathways, oxidative stress, and tumor immune cell infiltration. We also highlight the great diversity of miRNAs, lncRNAs, and circular RNAs which provide mechanisms for the ubiquitous tumor-specific suppression of KLF9 mRNA and protein. Elucidation of KLF9 and KLF13 in cancer biology is likely to provide new inroads to the understanding of oncogenesis and its prevention and treatments.

## 1. Introduction

**Figure 1:**
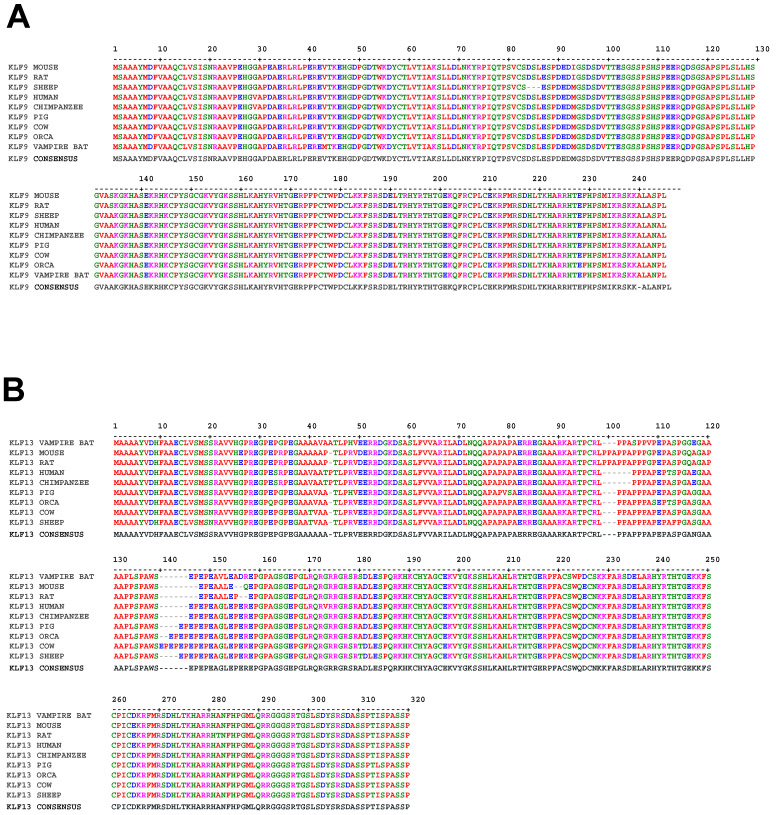
Multiple sequence alignment of mammalian KLF9 (panel (**A**)) and KLF13 (panel (**B**)) proteins. Human KLF13 protein has two isoforms; isoform 1’s sequence is shown (the protein that is described in the literature). KLF13 isoform 2 has a carboxy-terminus distinct from that of KLF13 isoform 1 and contains only one zinc finger (https://www.ncbi.nlm.nih.gov/gene/51621, accessed on 3 August 2023). Sequence data were aligned using Multiple Sequence Comparison by Log-Expectation (MUSCLE) [6]. Colors: red—small (small + hydrophobic (incl aromatic—Y)); blue—acidic; magenta—basic—H; green—hydroxyl + sulfhydryl + amine + G residues.

Specificity proteins (SPs) and Krüppel-like factors (KLFs) constitute an evolutionarily conserved group of transcriptional regulators (the SP/KLF family). The founding member of this family was SP1, whose original cDNA cloning and sequencing (a tour de force for the time, 1987) revealed the presence of three contiguous Zn finger protein domains, each requiring a bound Zn^2+^ ion for DNA binding activity [1]. Human genes encoding nine SPs and seventeen KLF proteins were subsequently identified, with KLF18 likely being a pseudogene present in extant placental mammals [2,3,4]. The SPs and KLFs were numbered corresponding to their order of discovery. KLFs comprise three subgroups based on molecular phylogeny: group 1 (KLF3, KLF5, KLF6, KLF7, KLF8, KLF12), group 2 (KLF1, KLF2, KLF4, KLF15, KLF17), and group 3 (KLF9, KLF10, KLF11, KLF13, KLF14, KLF16) [5]. KLF9 and KLF13 together constitute a unique clade within group 3, are two of the smallest KLFs [4], are most similar to each other in overall protein sequence in human and mouse [3], and arose early in metazoan evolution [5]. Examination of protein sequences of KLF9 and KLF13 across a variety of mammals shows a high degree of conservation for KLF9 and slightly less so for KLF13 (Figure 1).

The focus on the SP/KLF family has dramatically increased over the last three decades. In keeping with this robust increase, a number of excellent reviews have surveyed the entire field of KLFs with respect to their participation in normal and patho-physiology. These have included surveys/analyses of all KLFs and their known roles in: female reproductive system pathologies [3], digestive physiology and diseases [4], metabolic homeostasis and cardiac dysfunctions [7], energy metabolism [8], skeletal physiology and associated pathologies [9,10], adipogenesis [11], and oncology [3,4,12,13,14]. Recent reviews have focused solely on one KLF member and on one (or a few) aspect of normal physiology or pathophysiology recent examples [15,16,17]. The present review, in contrast, concentrates on the paralogous KLF9 and KLF13, their known roles in the suppression or promotion of cancer development, and the current gaps in knowledge regarding their respective biology, signaling, and oncogenic mechanisms.

## 2. General Aspects of KLF9 and KLF13

### 2.1. Selected Highlights in the KLF9/KLF13 Field

#### 2.1.1. KLF9

Rat and human *KLF9* (originally known as BTEB1) cDNAs (and corresponding protein) sequences were reported in 1992 and 1993, respectively [18,19]. A report of *KLF9* mRNAs in human T cell and macrophage/monocyte cell lines also appeared in 1993 [19,20]. In 1995, two regions of KLF9 required for its transactivation of the SV40 early gene were localized to amino acids 13–27 and 58–68 [21]. In 1997, *KLF9* expression was reported in the porcine female reproductive tract and primarily localized to the epithelial rather than stromal cells, reflecting cell type specificity for this expression [22]. In 1999, nuclear KLF9 was reported to be a functional partner of the nuclear receptor for the steroid hormone progesterone (PR) in controlling the pregnancy-associated, epithelial gene programs requisite for fetal development and pregnancy success [23]. Also in 1999, KLF9 was reported to be rapidly and highly induced by thyroid hormone in rat brain, thus linking KLF9, thyroid hormone action, and neurodevelopment [24]. Mice null for *Klf9* were reported in 2003 and were found to exhibit deficits in certain behavioral indices, linked to the absence of the expression of KLF9 in the developing cerebellum, hippocampus, and amygdala [25]. The *Klf9* knockout mice were a corresponding knock-in of the β-galactosidase-encoding sequence, and x-gal (5-bromo-4-chloro-3-indolyl-β-D-galactoside) staining of tissue whole mount preparations showed significant *Klf9* promoter activity in the liver, kidney, heart, brain, and bones [25]. A later study reported a sub-fertility defect in female *Klf9* knockout mice which resided at the level of the uterus, and which aligned with the cooperative actions of KLF9 and PR [26]. More recent studies detailed the molecular roles of KLF9 in neurogenesis, memory, anxiety, and neural stem cell dynamics [27,28,29]. The first report linking KLF9 with oxidative stress was published in 2014 [30]; this paper opened a now burgeoning subfield of KLF9 biology. The first hints of any kind of tumor-suppressive function for KLF9 were reported in 2008 and 2011; in these reports, KLF9 mRNA and protein levels were shown to be significantly reduced in human endometrial tumors when compared to adjacent uninvolved endometrium [31,32].

#### 2.1.2. KLF13

Human and mouse *KLF13* cDNAs (and corresponding protein sequences) were reported by several laboratories in 1999–2000; these reports described ubiquitous tissue expression of *KLF13* mRNAs [33,34,35]. The modular structure of KLF13 was demonstrated in 2002 [36] and mimicked that of KLF9. In human T lymphocytes, KLF13 was shown to be a specific transcriptional inducer of the gene encoding the chemokine CCL5 (C-C Motif Chemokine Ligand 5, previously known as RANTES) [33,36], a chemoattractant for monocytes, T cells, and eosinophils. Regarding human *CCL5* gene promoter induction by KLF13, CTCCC was shown to be the critical cis element [36]. Descriptions of KLF13 essential functions in vascular smooth muscle cells [37] and erythroid cells [38,39] followed in 2003 and 2005. In 2006 and 2007, the Nemer lab demonstrated the important role of KLF13 in cardiac gene expression and heart development [40,41]. *Klf13* knockout mice were initially described in 2007 and 2008 [42,43,44]. Studies using this mouse model implicated KLF13 in B and T lymphocyte development and function and erythropoiesis [42,43,44,45,46]. More recently, KLF13-associated mutations were shown to cause congenital heart disease [47,48]. The linkage of KLF13 to oncogenesis was first reported in 2010 in oral squamous cell carcinoma, where it was reported to be tumor promoting [49].

### 2.2. Oncogenic Features of KLF9 and KLF13

*KLF9* and *KLF13* genes, while ubiquitously expressed, manifest variable expression among tissues. For *KLF9* mRNA, the strongest tissue expression is demonstrated in brain, kidney, lung, and testis [18]. By contrast, *KLF13* mRNA showed the strongest expression in the lymph nodes, thymus, and heart [33]. Both genes are widely co-expressed during mouse fetal development, although some degree of cell-type- and tissue-specificity exists. For example, *Klf13* (but not *Klf9*) is highly expressed in the developing mouse heart, whereas in the developing gut and bladder, both genes are co-expressed (with *Klf13* expression being primarily epithelial, and *Klf9* expression being epithelial and muscular) [50].

Both KLFs are implicated in immune cell development and function, having a clear bearing on their roles in tumor development and the tumor microenvironment. KLF9 is relatively abundant in T cell- and macrophage/monocyte cell lines [20]. Moreover, KLF9 promotes hematopoiesis and T lymphopoiesis in zebrafish [51], negatively regulates B cell proliferation [52,53], and is positively associated with tumor immune cell infiltration [54]. As mentioned earlier, KLF13 is central to the transcriptional regulation of lymphocyte development and survival, and erythropoiesis. While not as extensively studied as family member KLF4 for functional contributions to stem cell biology, both KLFs 9 and 13 have been linked to normal stem cell expansion and maintenance, and the mechanistic regulation of cancer stem cell phenotypes [55,56,57]. Interestingly, in the standard NIH 3T3 transformation assay, KLF13, but not KLF9, suppressed oncogenic KRAS-mediated foci formation [58].

**Figure 2:**
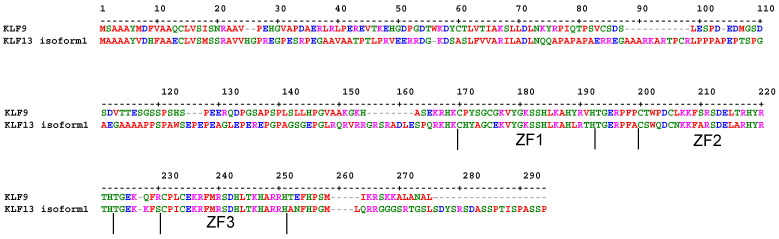
Multiple sequence alignment of human KLF9 and KLF13 isoform 1 proteins. The boundaries of the three zinc fingers are indicated [61]. Sequence data were aligned using Multiple Sequence Comparison by Log-Expectation (MUSCLE) [6]. Colors: red—small (small + hydrophobic (incl aromatic—Y)); blue—acidic; magenta—basic—H; green—hydroxyl + sulfhydryl + amine + G residues.

Where examined, KLF9 and KLF13 typically exert more repressive than inductive effects on gene transcription. However, one report demonstrated that KLF9 could function as an activator or repressor for the same gene (i.e., Fibroblast Growth Factor Receptor 1, (FGFR1)) via the same cis element, depending on the differentiation state of the cell (myoblast vs myotube) [59]. The consensus DNA sequence motif for KLF binding is 5′-C(A/T)CCC-3′; an interaction that is primarily mediated via the carboxy-terminal tripartite zinc finger (ZF) region (ZF1 and ZF2 = 23 amino acids, ZF3 = 21 amino acids) of each KLF (Figure 2). The more variable amino-terminus and carboxy-terminus of each KLF mediates trans-activation and trans-repression, by utilizing bound co-repressors, co-activators, and chromatin modifiers. For C_2_H_2_ zinc finger-containing proteins in general, orthology alone appears poor at predicting DNA binding elements [60]; thus, the actual cognate cis elements and corresponding chromatin binding sites for KLF9 and KLF13, in most cell and tissue contexts, remain unknown.

It is worth noting that mRNAs derived from both the *KLF9* and *KLF13* genes exhibit cell-specific translational regulation via sequences in their 5′-untranslated regions, sometimes leading to discordance between mRNA and protein levels [62,63]. In the cases of KLF9 and KLF13, this discordance has not been thoroughly examined for numerous cell or tissue types (including cancers), but this possibility should be considered when assigning function based solely on assays of mRNA abundance.

### 2.3. Hormonal and Other Regulators of KLF9 and KLF13 Genes

*KLF9* gene expression is rapidly and highly induced by thyroid hormone (via its nuclear receptors) in neuronal cells within the developing nervous system [24,64,65,66,67,68] and in hematopoietic cells and tissues [51]. However, thyroid hormone regulation of the *KLF13* gene has not been reported.

Glucocorticoids (via their nuclear receptors) are also potent and rapid inducers of the *KLF9* gene in Xenopus, mouse, and rat brains [28,69,70,71]; the developing lungs of Xenopus [72]; pulmonary epithelial cells [73,74]; liver [75]; macrophages [76,77]; keratinocytes [78,79]; and zebrafish larvae [80]. In human HEC-1-A endometrial cancer cells, stable transfection with KLF9 caused the induction of the *NR3C1* gene (Glucocorticoid Receptor, GR), suggesting the cross-regulation of KLF9 and GR [31]. Similarly, two other studies have found the inductive capacities of glucocorticoids and their receptors on the *KLF13* gene [81,82].

Progesterone, acting through its receptor, was reported to modestly increase *KLF9* gene expression in uterine endometrial cells [83,84]. By contrast, the transcription factor HOXA10, whose expression is subject to positive regulation via ovarian estrogen and progesterone, was demonstrated to decrease endometrial *KLF9* mRNA levels [85]. Conversely, KLF9 was reported to suppress androgen receptor (AR) expression in prostate cancer cells, highlighting its growth inhibitory effects on androgen-dependent tumor cells [86].

### 2.4. KLF9 and KLF13 Post-Translational Modifications and Interactomes

**Figure 3:**
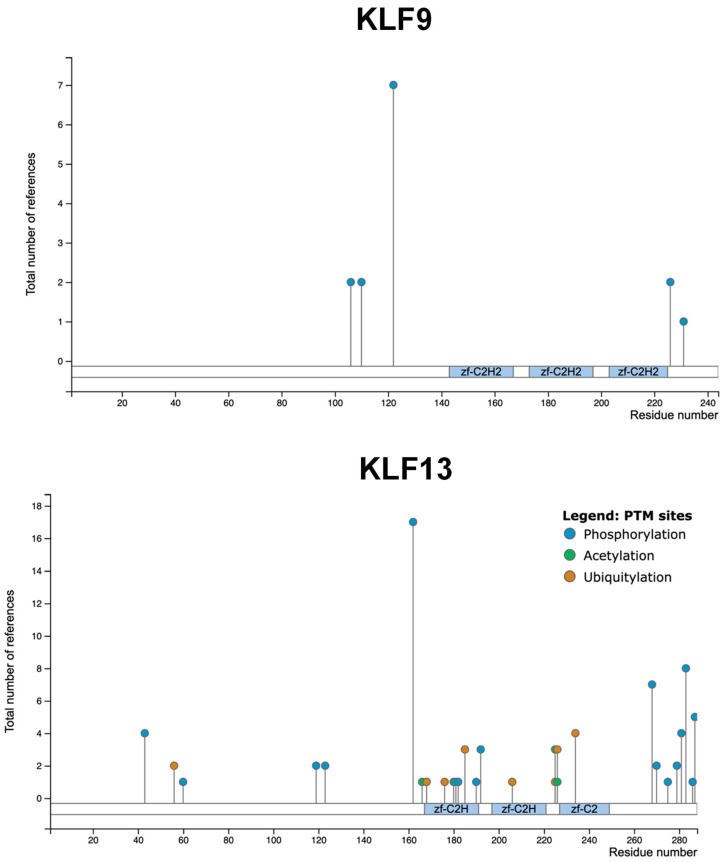
Post-translational modifications (serine and threonine phosphorylation, lysine acetylation, lysine ubiquitylation) mapped on human KLF9 and human KLF13 proteins. Data are from PhosphoSitePlus (v6.7.1.1) (https://www.phosphosite.org/homeAction.action, accessed on 3 August 2023), representing a collation of data from multiple targeted and mass spectrometry-based proteomics studies. The y-axis shows the relative strength (i.e., experimental evidence) of the presence of a site modification (note: the data are not restricted to cancer states, but also include that for normal tissues and other pathophysiological states). Zf-C2H: demarcates each of the three zinc fingers.

Until recently, both KLF9 and KLF13 suffered from a dearth of functional information regarding post-translational modifications (i.e., phosphorylation, acetylation, ubiquitylation) and functional/physical interaction partners [87,88]. However, of the PhosphoSitePlus database (a collation of data from targeted and mass spectrometry (MS)-based approaches) (https://www.phosphosite.org/homeAction.action, accessed on 31 August 2023) revealed a large number of serine and threonine residues in human KLF13 that are subject to phosphorylation, and a number of lysine residues subject to ubiquitylation or acetylation, including in the context of cancer(s) (Figure 3). KLF9, in contrast, had much fewer reported site modifications (Figure 3). The distinct modification patterns for KLF9 and KLF13 are striking and may underlie their discrete functionality. For example, the zinc-fingers-encompassing region of KLF9 is devoid of post-translational modifications, whereas for KLF13 it is highly modified (Figure 3).

In differentiating oligodendrocytes, KLF9 and KLF13 physically and functionally interact with each other to trans-activate target genes [89]. KLF9 and KLF13, along with KLF10, KLF11, KLF14, and KLF16, share a domain (separate from their DNA-binding domains) that binds the co-repressor SIN3A (SIN3 Transcription Regulator Family Member A) [7,90]. A functional interaction between KLF13, SIN3A, and HDAC1 (Histone Deacetylase 1, also a co-repressor) was first described in 2001 [91]. TGF-β is an inducer of KLF13 expression in certain cell and tissue contexts [92]. In a study utilizing kidney tubular epithelial cells, KLF13 was shown to physically interact with SIN3A and HDAC1 to suppress the transcription of TGF-β-response genes [92]; the latter reinforcing the connections and regulatory loops involving TGF-β, KLF13 (and its co-repressors SIN3A and HDAC1), and downstream gene targets.

In breast cancer cells, KLF9 recruits HDAC1 to the MMP9 (Matrix Metallopeptidase 9) gene promoter to repress MMP9 synthesis and consequent cell migration/invasion [93]. Other known interactors of KLF9 include Progesterone Receptor B (PR-B) [94] and ZZEF1 (Zinc Finger ZZ-type and EF-hand Domain Containing I, a histone reader) [95]. Interactors of KLF13 include CBP (CREB-binding Protein) and PCAF (P300/CBP-associated Factor) [87] and PRP4 (PRP4 Kinase) [96]. CBP and PCAF elicit divergent effects on KLF13 transcriptional activity via their acetylation of KLF13 in lysine residues within the DNA-binding domains [87]. PRP4, in contrast, phosphorylates KLF13, thereby affecting its DNA binding affinity, nuclear localization, and transcriptional activity [96]. KLF13 constitutes a paradigm for how a lynchpin member of an enhanceosome works at the level of a target gene [97,98]. Interactions with SIN3A and SIN3B likely explain, in part, the effects of cellular growth status and growth regulation, as well as the impacts of other signals and signaling pathways, on the transcriptional activity of both KLF9 and KLF13 [99,100,101,102]. For example, KLF9 physically interacts with JNK3 (Mitogen-activated Protein Kinase 8) to suppress axon growth in vitro and in vivo [88]. KLF13 interacts with TBX5 (T-box Transcription Factor 5) to induce cardiac genes [103].

More recently, high throughput screens (example, [104]) have identified many additional proteins interacting with KLF9 (https://www.ncbi.nlm.nih.gov/gene/687#interactions, accessed on 31 August 2023) and KLF13 (https://www.ncbi.nlm.nih.gov/gene/51621, accessed on 3 August 2023). However, no or little follow-up information is available to confirm their respective contributions to KLF9 or KLF13 functioning. As expected from their distinct patterns of post-translational modifications (Figure 3), KLF9 and KLF13 do not exhibit considerable overlap in their respective newly identified potential interacting partners. HNRNPH1 (Heterogeneous Nuclear Ribonucleoprotein H1) is one interactor that is shared by both KLF9 and KLF13, although its physiological significance to the functioning of either KLF is unknown. Based on the high throughput screens, KLF9 interacts with more than eighty other proteins, many of which are nuclear proteins with their own activator or repressor functions (e.g., ARID2 (AT-rich Interaction Domain 2), ARD4A, ARD4B, BRD7 (Bromodomain Containing 7), CTCF (CCCTC-binding factor), DDX10 (Dead-box Helicase 10), DDX18, DDX24, DDX27, and YY1 (YY1 Transcription Factor)). This information constitutes a potential goldmine for the exploration of higher order complexes, involving KLF9 and KLF13, that might regulate chromatin structure and gene accessibility during oncogenesis.

### 2.5. Epigenomics of KLF9 and KLF13

Of the two proteins, KLF13 was the first to be implicated in the regulation of chromatin structure in the promoter/enhancer regions of target genes [98]. An examination of a current DNA methylation database (MethHC 2.0) indicates that *KLF9* and *KLF13* gene promoter regions are over- or under-methylated in small subsets of human cancers (Table 1). Strikingly, with the exception of thyroid cancers, the *KLF9* promoter region was more methylated in tumors than in corresponding normal tissues from the colon, esophagus, kidney, liver, and lung. By contrast, the *KLF13* promoter region was less methylated in tumors than in corresponding normal breast, bile ducts, liver, adrenal gland, and endometrium, and more methylated in kidney tumors than corresponding normal tissue (Table 1). These data support a more general role for KLF9 as a tumor suppressor, whereas KLF13 may be a tumor suppressor or promoter, depending on the tissue.

Reports of differentially methylated regions of the *KLF9* gene and their respective consequences on physiological processes are infrequent, but nevertheless do exist [107,108,109,110]. These reports mainly concern CpG-methylation of the *KLF9* gene and the effects on/associations with thyroid hormone status and childhood obesity. *KLF9* is one of a number of genes that are induced in MDA-MB-231 cells by co-treatment with the DNA methyltransferase (DNMT) inhibitor 5-aza-2′-deoxycytidine and the HDAC inhibitor suberoyl anilide bis-hydroxamide [111]. Similarly, in human colorectal cancer cell lines, use of 5-aza-2′-deoxycytidine revealed *KLF9* as a methylation-silenced gene [112]. Recent studies identified a site of CpG methylation within the *KLF13* gene that was significantly associated with body mass index, obesity, and appetite regulation [113,114]. Lastly, DNA Methyltransferase I (DNMT1)-mediated hypermethylation of the *KLF13* gene promoter with correspondent down-regulation of gene expression in glioma has been reported [115].

### 2.6. Redundancy, Overlapping Actions, and Functional Networks of KLF9 and KLF13

Emerging evidence indicates that KLF9 and KLF13 functionally overlap and/or compensate for each other to varying degrees, participate in KLF networks, and exhibit cross-regulation, albeit in tissue-specific contexts [3,89,116,117,118,119,120,121,122,123]. KLF9 and KLF13 can functionally compensate for each other, at least partially, with respect to female reproduction physiology, regulation of the mammalian circadian clock, hippocampal function, and oligodendrocyte differentiation [89,116,117,118,119,120,122,123]. In the studies by Avila-Mendoza et al. [123,124] on HT-22 neuronal cells, KLF9 and KLF13 overlapped in the binding sites of certain gene promoters, while other genes had only KLF9 or KLF13 association. As an example, the suppression of *KLF16* mRNA expression by both KLF9 and KLF13 was associated with their respective binding to the *KLF16* gene promoter region. It remains unknown, however, whether such associations, of KLF9 and KLF13 with the same gene (e.g., *KLF16*), occur in cis or as a complex.

One important physiological context for cancer pre-disposition in which KLF9 and KLF13 are known to intersect in function is adipogenesis. Both transcription factors are pro-adipogenic and partially overlap with respect to the mechanism(s) of action in this regard [11]. *KLF9* and *KLF13* gene variants (SNPs, methylated CpG) are associated with body mass index [113,114,125]. Further, both KLF9 and KLF13 promote adipocyte differentiation, in part by transactivating the *PPARG* gene [126,127], the latter encoding Peroxisome Proliferator Activated Receptor Gamma (PPARγ), a master regulator of adipogenesis. Since adipogenesis underlies obesity and consequent adipocytokine secretion, and several adipocytokines are cancer-promoting (e.g., leptin), KLF9 and KLF13 may significantly affect cancer pre-disposition indirectly through their targeted actions on fat depots. However, this hypothesis has not been examined experimentally.

### 2.7. Known Pathways Subserving KLF9 and/or KLF13 Actions

KLF9 and KLF13 exert pro- or anti-proliferative and pro- or anti-apoptotic effects, depending on cell and physiological contexts [42,68,77,128]. Several major pathways are known to subserve KLF9 and KLF13 actions [128,129]. A well-implicated pathway in both KLF9 and KLF13 is the mediation of cAMP signaling. KLF9 and KLF13 repress mouse cAMP-dependent hippocampal neurite outgrowth in vitro, in part by repressing expression of multiple genes encoding participants in this signaling pathway [123]. For these cells at least, KLF13 was demonstrated to be more potent than KLF9, a consequence of the ~4-fold higher numbers of target genes being affected by KLF13 than by KLF9. Convergence of cAMP signaling and KLF9 also was observed during the transition of primordial germ cells to pluripotent stem cells [57].

Similarly, both KLF9 and KLF13 are known to participate in TGF-β pathways. Over-expression of KLF9 in the endometrial carcinoma cell line HEC-1-A rendered these cells more proliferative to TGF-β [128]. Acute TGF-β treatment of renal tubular epithelial cells caused a rapid induction of *KLF13* gene and protein, resulting in the inhibition of TGF-β target genes via the formation of a co-repressor complex consisting of KLF13, SIN3A, and HDAC1 [92].

Another pathway that integrally utilizes KLF9 and KLF13, and which has potential implications in oncogenesis and cancer therapy, is the circadian clock [130,131,132,133]. The links with the circadian clock and KLF9 were initially explored in human keratinocytes [78]. In this context, oscillations in *KLF9* expression were shown to be driven by rhythmic variations in systemic cortisol levels, which, in turn, resulted in a rhythmic repression of KLF9 target genes, including those that drive proliferation. Similar observations have been made in human breast cancer cell lines [133]. *KLF9* and *KLF13* genes also exhibit rhythmic mRNA expression in the mouse liver [122,134], but in the mouse hippocampus, only *KLF9* mRNAs exhibit oscillations [122]. In chromatin, KLF9 and KLF13 proteins are associated with multiple core clock and clock-output genes. In HT22 cells, a single knockout of *KLF9* or *KLF13* did not affect the expression of the clock-output gene Dbp, whereas a double-knockout of *KLF9* and *KLF13* disrupted the oscillatory expression of Dbp [122]; the latter an example of functional redundancy of KLF9 and KLF13. Recently, the oscillatory behavior of KLF9 has been explored within the context of KLF9 suppression in breast cancer [133]. By contrast, KLF13 has not been implicated to a comparable degree to the nexus of circadian physiology and oncogenesis.

FBW7 (F-box and WD Repeat Domain-containing 7), a component of the ubiquitylation pathway(s), is a tumor suppressor for multiple human cancers [135] and a negative regulator of steady-state levels of KLF13. A possible link between KLF13 and FBW7 in carcinogenesis is suggested by the findings that FWB7 is downregulated during the HPV life cycle while KLF13 levels become elevated in pre-neoplastic cervical epithelium [136].

### 2.8. KLF9 and KLF13 in Oxidative Stress (OS)

KLF9 has increasingly become a relevant participant in the promotion and/or inhibition of OS in cells, tissues, and organisms. KLF9 is induced by threshold levels of OS in multiple types of cells via the transcription factor NFE2L2 (NFE2 Like bZIP Transcription Factor 2), the latter mediating the canonical antioxidant pathway [30]. In mouse and human fibroblasts as well as melanoma, colon, and breast cancer cell lines, the OS-induced expression of KLF9 leads to a further increase in intracellular ROS, as a consequence of KLF9 suppression of Thioredoxin Reductase 2 (TXNRD2; a key anti-oxidant protein) gene expression [30]. The end result is augmented apoptosis as a consequence of unopposed high levels of OS. This seminal work was followed by multiple reports describing the association of KLF9 with the repression of other antioxidant genes/proteins, catastrophic oxidative stress, and apoptosis [77,137,138,139,140,141,142,143,144,145,146]. In marked contrast, the absence of the *Klf9* gene in mice fed obesogenic diet caused increased (rather than decreased) OS in liver and serum [147]. Similarly, the absence of KLF9 in endometriotic lesions in a mouse model of endometriosis, led to increased OS both systemically and in lesions [148,149].

Currently, there is limited evidence supporting the role of KLF13 within the pathways of OS and anti-OS. A single report described the role of KLF13 in protecting mouse cardiomyocytes from DNA damage and cell death after treatment with CoCl_2_ or doxorubicin and the associated OS [82].

KLF9 also is implicated in proteotoxic stress [150] as well as ER stress and the cytotoxic unfolded protein response [139,151]. Thus, there is a pattern emerging for the associations of KLF9 with cell, tissue, and somatic stress responses: the latter are themselves implicated in cancer development, as well as in determining the efficacy of cancer treatments. This conclusion is further buttressed by several reports on the associations/links between KLF9 (but not KLF13) and Jun N-terminal Kinase (JNK) pathway(s) [88,129,152].

### 2.9. KLF9, KLF13 and Tissue Fibrosis

KLF9 and KLF13 are implicated in fibrosis, a pre-disposing condition for malignancy in several tissues, albeit in opposing manners. KLF9 promotes fibrosis via its induction of alcohol (acetaldehyde)-induced COL1A1 (Collagen Type I Alpha I Chain) gene expression in rat liver stellate cells [152]. By contrast, KLF13 has an anti-fibrotic function in the lung [153] and kidney [92].

### 2.10. KLF9 and Cancer Stem Cells

KLF9 (and to a lesser degree KLF13) represses the cancer stem cell phenotype in glioblastoma and ovarian cancer, and thus, perhaps other cancers as well [154,155,156,157,158]. In glioblastoma and ovarian cancer, KLF9 was shown to repress the expression of the Notch 1 gene, thereby resulting in reversion away from the stem cell phenotype due to less active Notch signaling [154,157].

## 3. Cancer-Specific Aspects of KLF9 and KLF13

The known associations of KLF9 and KLF13 with cancers in humans (and relevant mouse models) are summarized below. As is typical, these results were generated from studies utilizing knock-down and/or over-expression of the KLF gene/protein in cancer cell lines, followed by measurements of proliferation, apoptosis, migration, and epithelio-mesenchymal transition (EMT). Experiments conducted in parallel confirmed the actions of specific miRs, lncRNAs, and circRNAs on KLF9 or KLF13 protein abundance and consequent effects on tumor cells in vitro and in vivo. Further, the tumoral expression of *KLF9* and *KLF13* mRNA and/or proteins was compared to the corresponding adjacent normal tissue.

### 3.1. Bladder Cancer

Knock-down of *KLF9* increased proliferation and colony formation of human bladder cancer cell lines. Moreover, the oncogenic miR-636, targeting the 3′-UTR of *KLF9* mRNA, inhibited *KLF9* expression in these cancer lines [159].

### 3.2. Breast Cancer

*KLF9* gene and protein expression is lower in breast cancers when compared to paired normal tissue; moreover, KLF9 inhibited breast cancer cell proliferation and invasion in vitro and in vivo [93,107,133,160,161,162,163]. Part of the suppressive effect of KLF9 on breast cancer promotion was suggested to involve its role in repressing E-cadherin expression [93] and in supporting normal circadian physiology [133].

### 3.3. Cervical Cancer

Cervical cancer patients exhibit lower blood levels of *KLF9* mRNA compared to healthy non-cancerous controls, suggesting the possible utility of KLF9 as a non-invasive biomarker for this cancer type [164]. However, similar findings have been limited for other cancers, with the exception of multiple melanoma [165]. In contrast, *KLF13* is over-expressed in cervical cancer cell lines, and *KLF13* expression in normal cervical epithelium is low but increases in intraepithelial cervical neoplasia and invasive squamous cervical cancer. The latter is consistent with the suggested participation of KLF13 in the HPV life cycle [136].

### 3.4. Colorectal Cancer

*Klf9*-null mice have an increased number of goblet cells and a tendency for decreased crypt depth in their colons, pointing to the growth, as well as differentiative, roles of KLF9 in this tissue [55]. In the classical Apc^Min/+^ mouse model of intestinal cancer, KLF9 immunoreactivity was low to undetectable in adenomas of the ileum, but was detected in the crypts and villus lamina propria cells of the adjacent normal-appearing mucosa [166]. In another report, *KLF9* mRNA and protein was significantly less abundant in human colon adenomas and adenocarcinomas than in the corresponding normal colon mucosa [167]. In this study, KLF9 immunoreactivity was localized to the crypt bases in normal human colons. Brown et al. (2015) [168] reported the significant down-regulation of *KLF9* mRNA abundance in human colon and rectal cancers, in agreement with the above earlier report [167]. Subsequently, differential tumoral vs. normal *KLF9* mRNA expression was shown to be a predictor (in combination with several other genes) of the overall survival of CRC patients [169]. The circRNA circNOL10 has been reported to sponge (bind) miR-135a-5p and miR-135-5p, both of which, in turn, can bind *KLF9* mRNA, thereby resulting in an overall inhibition of proliferation, migration and invasion of CRC cells in vitro [170].

Heterozygous and homozygous knockouts of the *Klf9* gene in the background of the Apc^Min/+^ mutation led to significantly more colon adenomas in both male and female mice, further supporting the colon tumor-suppressive role of KLF9 [168]. In this study, gene expression profiling of colon mucosa, at a timepoint prior to the overt appearance of adenomas, revealed the induction of a subset of interferon-inducible genes (including CXCL9 (CXC Motif Chemokine Ligand 9) and ISG15 (Interferon-stimulated Gene 15)) upon heterozygous and homozygous knockout of the *Klf9* gene (in the Apc^Min/+^ background [168]. The inhibitory effect of KLF9 on IFN-induction of these genes was recapitulated in vitro using a human colorectal cancer cell line (HT-29) [168]. While this repressive effect of KLF9 has, so far, been demonstrated solely for this cancer type, the findings may be relevant to other cancer type(s) and other KLF family members. In this regard, in the grouper (an ocean fish) KLF9 protein suppresses the expression of several interferon-inducible cytokines, including ISG15 and IFNγ in a grouper spleen cell line [171], demonstrating that this immunological role for KLF9 is conserved across phylogeny. KLF4 (a well-established tumor suppressor in the gut) is induced by IFNγ in the HT-29 cell line, where it inhibits proliferation and induces apoptosis [172]. KLF4, in turn, represses the expression of the interferon-induced transmembrane (IFITM) protein IFITM3, a gene that is overexpressed in human colorectal cancers and which contributes to tumor growth and metastasis [173]. Another report identified KLF5 as an inducer of *IFITM1*, *IFITM2,* and *IFITM3* genes in A549 (human lung carcinoma cell line) [174]. Thus, there is precedence for a potentially wider convergence of KLFs with interferon-induced proteins and pathways in cancers.

In a previous report [168], KLF9 was shown to repress PD-L1 (*CD274*) gene expression in the neoplastic colon mucosa of *Klf9* KO, Apc^Min/+^ mice, and in human HT-29 CRC cells. These observations are intriguing, especially in view of the current immune therapies targeting PD-1/PD-L1, as well as the reported independent correlations of PD-L1 immunopositivity in colorectal tumor cells and conversely PD-L1 immunonegativity in colorectal tumor-infiltrating lymphocytes, with worse overall survival of CRC patients [175]. Further investigations in this direction are required to support the therapeutic value of monitoring of KLF9 expression.

The literature regarding KLF13 and colorectal cancer is much less robust than that for KLF9. Nevertheless, reduced colon tumoral expression of *KLF13* mRNA (compared to normal corresponding tissue) in humans has been described [176], similar to that for *KLF9*. A second study confirmed the lower *KLF13* mRNA abundance in human colorectal tumors than in corresponding normal tissue [177]. This same study demonstrated the potent suppressive effects of KLF13 on the proliferation and colony formation of colorectal cancer cells, on their tumor formation in nude mice, and on tumor cell cholesterol biosynthesis.

### 3.5. Cutaneous Squamous Cell Carcinoma

In A431 tumor cells, KLF9 is a repressor of the PFKFB3 (6-Phosphofructo-2-kinase/fructose-2,6-biphophatase 3) gene, which encodes a protein that stimulates proliferation and metastatic behavior in vitro [178]. In vivo studies confirming the above results are currently lacking.

### 3.6. Endometrial Cancer

Given that endometriosis predisposes patients to cancers of the female reproductive tract [179,180], we first summarize the current state of knowledge on the involvement of KLF9 and KLF13 in this condition. In women with endometriosis, mRNA abundance of *KLF9* in eutopic endometrium was lower than for women without endometriosis [181]. In a mouse model of endometriosis, the absence of KLF9 promoted ectopic endometrial lesion establishment [182]. In this study, endometriotic lesions devoid of KLF9 had activated Notch and Hedgehog signaling and attenuated progesterone receptor expression. The associations between diminished KLF9 expression, the activation of Notch signaling, and a loss of progesterone receptor protein levels in endometriotic lesions were confirmed in women with endometriosis and mechanistically in vitro using human endometrial stromal cells [183]. *KLF13* transcript levels also were of lower abundance in eutopic endometrium of women with endometriosis when compared to non-diseased tissue [120]. In a mouse model of endometriosis, the absence of a functional *KLF13* gene had no effect on endometriotic lesion incidence, volume, and number, or proliferative and apoptotic status [120]. However, mouse endometriotic lesions lacking KLF13 had decreased expression of progesterone receptors as well as diminished estrogen receptor-α (ESR1) expression and signaling [120]. Interestingly, *KLF13*-null lesions had no alterations in activity or readout in the Notch or Hedgehog signaling pathways when compared to corresponding wildtype lesions, indicating that KLF13 had less involvement than KLF9 in the pathogenesis of endometriosis [120]. Nevertheless, the above studies reinforce the links between KLF9 and KLF13 and estrogen and progesterone signaling, which likely go awry during the transition from normal endometrium to a pathological state. In this regard, endometrial cancers are associated with unopposed estrogen activity.

Endometrial tumors had markedly reduced levels of *KLF9* mRNA and protein when compared to paired normal endometrial tissue [31,32]. Two additional studies confirmed these earlier findings [84,184]. Conversely, *KLF13* mRNA abundance was elevated in endometrial tumors vs. normal endometrium [32]. Over-expression of KLF9 in the human HEC-1-A endometrial cancer cell line implicated KLF9 in signaling TGF-β and in the induction of p21 (CDKN1A) (a tumor suppressor) [128]. This same in vitro context identified positive links between KLF9 and the PKA and JNK pathways, and corresponding signaling in endometrial carcinoma HEC-1-A cells [129]. KLF9 is a repressor of estrogen receptor α actions on select nuclear target genes in Ishikawa endometrial cancer cells and this ability may contribute to aberrant cell growth responses in the face of ovarian estrogen stimulation, when KLF9 expression is lowered or absent [185,186]. Indeed, the treatment of human Ishikawa endometrial cancer cells with estrogen stimulated their proliferation in vitro, an effect that was enhanced by the knock-down of KLF9 [32]. In another study, KLF9 expression was inversely associated with endometrial cancer metastasis, which involved, in part, the inhibition of Wnt/β-catenin signaling [84].

### 3.7. Esophageal Squamous Cell Carcinoma (ESCC)

Expression of the *KLF9* gene and protein was lower in ESCC tumors than in paired normal tissue [187]. In addition, over-expression of KLF9 in ESCC cells inhibited their proliferation, colony formation, and migration in vitro, and their metastatic potential in vivo [187].

### 3.8. Gastric Cancer (GC)

With respect to gastric cancers, KLF9 is reported to be stimulatory [188] or inhibitory [189] to cancer cell proliferation and their invasive ability. Interestingly, in the latter paper, KLF9 repressed gene expression of MMP28 (Matrix Metallopeptidase 28), an example of KLF9 trans-repression of a gene supporting metastasis. KLF9 was reported to have a lower expression in GC than in paired normal tissue, and this down-regulation correlated with GC cell metastasis in vitro and in vivo [189]. KLF13 also is less expressed in GC than paired normal tissue, inhibited the proliferation of GC cells in vitro, and suppressed the growth of GC xenografts in vivo, which were mechanistically related, in part, to the degradation of β-catenin [190].

### 3.9. Gliomas and Glioblastoma (GBM)

*KLF9* expression is markedly reduced in gliomas [191,192], and glioma patients with higher *KLF9* expression in their tumors survived longer than those with lower tumoral *KLF9* expression [191]. Forced expression of KLF9 inhibited glioma cell proliferation in vitro and tumor growth in vivo, in part, by suppressing expression of SOD1 (Superoxide Dismutase 1) and miR-21, the latter being a known pro-tumorigenic molecule [144,191]. miR-940 also is inversely associated with KLF9 mRNA, and interestingly, the knock-down of KLF9 attenuated the positive effects of miR-940 on glioma cell proliferation and invasion in vitro [192].

Over-expression of KLF9 suppressed glioblastoma cell stemness, in part, by repressing transcription of members of multiple signaling pathways that promote oncogenesis and stem cell phenotype, i.e., integrin, CXCR4 (C-X-C Chemokine Receptor Type 4), and Notch pathways [193]. Interestingly, the repression of ITGA6 (Integrin Alpha-6) and ITGA9 (integrin signaling pathways) in GBM cells by KLF9 resembles the repression of ITGB8 gene by KLF9 in endometrial cancer cells [31]; thus, KLF9 may broadly function as a repressor of specific cell–cell and cell–extracellular matrix interactions between cancer cells. In addition, KLF9 inhibits the transcription of the Notch1 gene in GBM neurosphere cells, resulting in the repression of Notch signaling and correspondingly reducing the expression of the Notch pathway genes *HES1* (Hairy and Enhancer of Split-1) and related *HES5* and *HEY2* genes [154]. Combining KLF9 over-expression with an HDAC inhibitor triggered synergistic cell death in GBM stem cell cultures [155]. In this latter study, the effects of KLF9 on catastrophic oxidative stress were not examined.

Like KLF9, KLF13 was shown to inhibit glioma cell proliferation and invasion in vitro, and moreover, patients with higher tumoral *KLF13* expression had a better prognosis than those with lower *KLF13* expression [115,156].

### 3.10. Hepatocellular Carcinoma (HCC)

KLF9 is abundantly expressed in liver cells where it is an important player in determining hepatic cell phenotypes, some of which occur in conjunction with thyroid hormone signaling [75,147,194,195,196,197]. Evidence suggests that KLF9 may be growth inhibitory for this organ during development in both the mouse and zebrafish [131,147]. KLF13 has been invoked, although to a lesser extent, in this tissue context [198]. The published evidence points to the more disparate than similar roles of KLF9 and KLF13 in HCC.

*KLF9* mRNA and protein levels are significantly lower in HCC tissue than in normal non-tumor liver tissue [199,200,201]. In HepG2 human liver cancer cells, the over-expression of KLF9 (by transfection) resulted in decreased proliferation and increased apoptosis [199,200]. In line with this, forced liver tumor expression of KLF9 in nude mice caused the regression of tumors accompanied by increased apoptosis and the decreased proliferation of xenografts [200]. In addition, a recent study found that over-expression of KLF9 caused suppression of metastasis of HCC tumor cells, whereas knock-down of KLF9 caused the opposite effects, ascribed, in part, to KLF9 repression of the EMT pathway [201]. KLF9 is a suppressor of oxidative stress and inflammation in the livers of high fat-fed mice [147], effects that are predicted to be tumor-preventive. It is tempting to speculate that the inhibitory effect of thyroid hormone on HCC development [202] involves, in part, the intermediate induction of *KLF9* gene expression.

*KLF13*, in contrast to *KLF9*, is over-expressed in HCC tissue compared to normal liver tissue, and the knock-down of *KLF13* caused inhibition of HCC cell proliferation, migration and invasion, and promotion of HCC cell apoptosis in vitro [203]. Conversely, over-expression of KLF13 stimulated HCC xenograft growth in nude mice, in part, via induction of HMGCS-1 (3-Hydroxy-3-Methylglutaryl-CoA Synthase 1), and thus enhanced synthesis of cholesterol [203] by the induction of the *ACOT7* (Acyl-CoA Thioesterase 7) gene (encoding a tumor-promoting protein involved in fatty acid metabolism) [204]. However, in another study, the knock-down of *KLF13* resulted in decreased apoptosis and increased proliferation of HCC cells in vitro [205]. It is presently unclear if the differences between the above studies concerning apoptosis and proliferation are due to different cell lines, or some other unknown variable(s).

### 3.11. Kidney Cancer (Renal Cell Carcinoma; RCC)

*KLF9* expression was significantly lower in RCC tissue compared to adjacent normal kidney tissue, and also in RCC compared to normal urothelial, cell lines [206]. Consistent with this, tumor cell levels of *KLF9* and *KLF13* are positive factors for overall survival of patients with clear cell renal cancer [207,208]. Moreover, miR-140-5p, which targets the 3′-UTR of *KLF9* mRNA, was greatly increased in RCC tumors and cell lines and promoted RCC proliferation, migration, and invasion [206].

### 3.12. Melanoma

In mouse models of melanoma, the absence of KLF9 was inhibitory to melanocyte proliferation, but had no effect on primary tumor growth while promoting metastasis [209].

### 3.13. Multiple Myeloma (MM)

*KLF9* mRNA levels in the sera of multiple myeloma (MM) patients are significantly lower than that for healthy subjects [165]. miR-135b-5p directly targets *KLF9* mRNA in MM cells, and the consequent repression of KLF9 expression favors increased proliferation and invasion in vitro [165]. KLF9 is a mediator of drug-induced apoptosis in MM plasma cells and its levels are induced in these cells by bortezomib in combination with an HDAC inhibitor [150].

### 3.14. Nasopharyngeal Carcinoma (NPC)

Unlike most cancer types, NPC tumors are reported to have significantly higher levels of *KLF9* mRNA than the corresponding normal tissue [210]. In these cells, miR-141-3p directly targets *KLF9* mRNA and the binding of the lncRNA SNHG15 to miR-141-3p, up-regulates KLF9 expression, and leads to the subsequent promotion of NPC oncogenesis [210]. This is a tissue example of the putative tumor-promoting effects of KLF9.

### 3.15. Neuroblastoma (NB)

Differentiated NB tumors manifest more KLF9 protein than do poorly differentiated NB tumors, with the latter type having a worse prognosis [211]. Overexpression of KLF9 in NB cell lines suppressed their proliferation and invasion in vitro, in part via the repression of the sonic hedgehog gene and its downstream signaling pathway [211].

### 3.16. Non-Small Cell Lung Cancer (NSCLC)

*KLF9* gene expression is markedly lower in NSCLC tumors than matched adjacent normal lung tissue [212,213], which may be partly due to oncogenic miR-570 targeting of the 3′-UTR of *KLF9* mRNA, as shown in tumor cell lines [212]. Similarly, overexpression of miR-141, which also targets the 3′-UTR of *KLF9* mRNA, enhanced the proliferation and invasion in vitro of a human lung cancer cell line, effects which were partially reversed by forced expression of KLF9 [213]. miR-889 appears to be yet another oncogenic miR that targets *KLF9* mRNA in NSCLC [214]. Other KLF9-targeting miRNAs with oncogenic properties so far identified in human lung cancer cells include mi-R-660-5p, miR-20a-5p, and miR-300 [215].

Similar to *KLF9*, lower expression of *KLF13* in NSCLC tumors has been associated with decreased overall patient survival [216], but the mechanistic underpinnings of KLF13 actions have yet to be elucidated.

### 3.17. Oral Squamous Cell Carcinoma (OSCC)

*KLF9* is over-expressed in human OSCC tumor tissue compared with adjacent normal tissue [217]. The tumor-associated increase in *KLF9* expression was ascribed to KLF9′s stimulation of LINC00664 gene activity, which has been previously shown to promote tumor growth in vitro and in vivo [217]. Similarly, the *KLF13* gene is overexpressed in OSCC cells and tumors, and siRNA knock-down of KLF13 in OSCC cells decreased their proliferation and radio-resistance.

### 3.18. Osteosarcoma (OS)

Knock-down of KLF9 in OS cell lines promoted their proliferation and invasion in vitro [218,219]. miR-378 and miR-652 negatively regulated KLF9 expression in OS cells [218,219]. miR-889, itself negatively regulated by circRNA_0078767, is also a negative regulator of KLF9 in OS cells [220]. A further mechanistic insight into KLF9′s role in OS was provided by a study showing that miR-338-3p, which suppressed the proliferation, migration, and invasion of OS cells in vitro, is transcriptionally induced by KLF9 [221].

### 3.19. Ovarian Cancer

Three papers have described the disparate actions of KLF9 in ovarian cancer. In the work of Zhang et al. (2015) [222], knock-down of *KLF9* in human ovarian cancer cell lines inhibited their proliferation, as well as their growth as xenografts in mice. Moreover, this paper reported that ovarian tumor tissue manifests higher expression of *KLF9* mRNA and protein than normal tissue. However, two other papers demonstrated the opposite: that KLF9 overexpression inhibited the ‘stemness’ phenotype as well as proliferative and metastatic abilities of ovarian cancer cells [157,158]. Aligned with these potential tumor repressive actions of KLF9 are the findings that miR-600, which targets the 3′-UTR of *KLF9* mRNA, is more highly expressed in ovarian cancers than in normal tissue, and elicited effects opposite to those of KLF9 in ovarian cancer cells [158]. Moreover, KLF9 inhibited Notch1 promoter activity in ovarian cancer cells and a loss of *KLF9* expression in tumors was predictive of worse patient prognosis [157,158].

### 3.20. Pancreatic Cancer

The significant roles of both KLF9 and KLF13 in suppressing pancreatic ductal adenocarcinoma (PDAC) formation have emerged. The earliest study in this area described the effect of KLF13, but not KLF9, on increasing the apoptosis of PANC1 cancer cells in vitro [58]. In that study, neither KLF had any effect on PANC1 cell proliferation. Three more recent studies reached a consensus that the expression of *KLF9* is significantly reduced in PDAC tumors (compared to normal tissue) and is inversely associated with patient survival [223,224,225]. Zhong et al. (2018) [225] reported the significant inhibitory effects of KLF9 on PANC1 proliferation (in contrast to the earlier work reported in Fernandez-Zapico et al. (2011)) [58] and on EMT, as well as the inductive effects of KLF9 on apoptosis. Another study described an inhibitory effect of KLF13 on EMT of PDAC cells [226]. The latter may occur via transcriptional targeting of lncRNA LINC00261 by KLF13, which has previously been shown to contribute to the suppression of migration/metastasis of PDAC cells [226].

### 3.21. Papillary Thyroid Cancer (PTC)

*KLF9* mRNA abundance is significantly lower in PTC tumors when compared to normal corresponding tissue, and the over-expression of KLF9 in PTC cell lines inhibited their proliferation, invasion, and migration [227].

### 3.22. Pedatric Acute Lymphoblastic Leukemia (ALL)

Glucocorticoids are part of combination chemotherapy for pediatric ALL. In glucocorticoid-sensitive, patient-derived xenografts (PDX), glucocorticoid receptor binding to the *KLF13* gene caused the induction of *KLF13* expression, which was followed by KLF13-elicited down-regulation of MYB gene/protein (an anti-apoptotic protein) in lymphoid cells [81]. However, the reciprocal changes in *KLF13* and *MYB* expression were not noted in glucocorticoid-resistant PDXs.

### 3.23. Prostate Cancer

KLF9 is a potent growth suppressor in prostate cancer cell lines in vitro and this involves, in part, the repression of two major pathways, namely those for AKT and the androgen receptor (AR), along with the induction of apoptosis [86,228]. *KLF9* knock-down caused an increase in prostate cancer cell spheroid formation, the latter an index of cancer stem cell phenotype [229]. Targeting by miR-141-3p of the 3′ UTR of *KLF9* mRNA may underlie tumor-mediated loss of KLF9 expression since this miRNA is induced in prostate tumors, and moreover, enhances sphere formation by prostate cancer cells in vitro [229].

Similar to *KLF9*, *KLF13* mRNA and protein levels are significantly lower in prostate tumors than in adjacent non-tumor tissue [230]. KLF13 is a suppressor of prostate cancer cell growth in vitro and of xenografts in mice, and like KLF9, suppresses AKT signaling and promotes apoptosis [230].

### 3.24. Testicular Seminoma

*KLF9* mRNA abundance was significantly reduced in testicular seminomas when compared to precancerous matched normal tissue, with the more advanced tumors displaying a further reduction in the levels of KLF9 mRNA [231]. Over-expression of KLF9 suppressed cell proliferation, cell migration, and cell invasion in vitro, effects functionally linked to KLF9 induction of miR-483-3p gene expression [231].

## 4. Gaps in Knowledge and Future Directions

**Figure 4:**
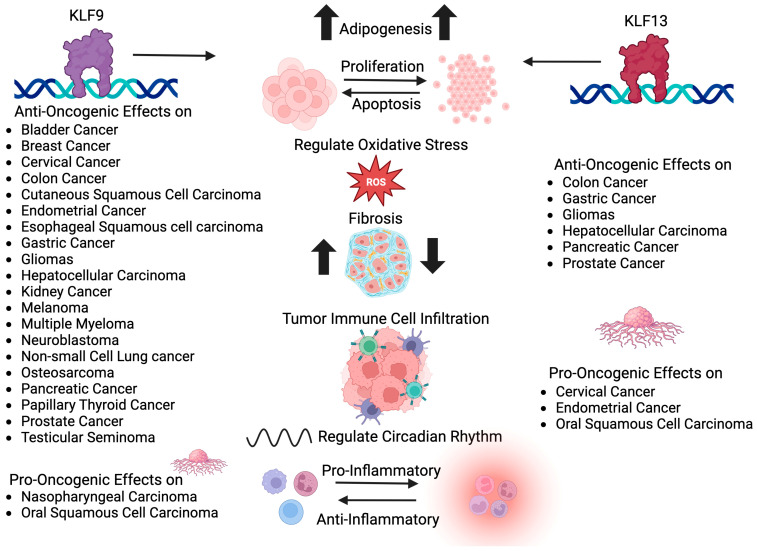
Summary and comparison of tumor-suppressing and tumor-promoting properties of KLF9 and KLF13. Created with BioRender.com.

The above narrative highlights the alterations (primarily reductions) in KLF9 and KLF13 steady state levels in many different cancers, and their predominant roles as suppressors or promoters of tumor cell growth and migration. The results make a strong case for further explorations of the mechanistic underpinnings of their tumor modulatory functions in multiple cancers (Figure 4). Below we expand on some questions ripe for further examination, with potential short- and long-term benefits to the understanding of, as well as drug discovery, treatment, and management of various malignancies.

While the field has a foundational comprehension of KLF9 and KLF13 expression and overall actions in cancer cell lines, there are likely to be significant, as yet unknown, variations in the cognate DNA sequence elements that subserve KLF9 and KLF13 actions in normal, pre-cancerous, and cancerous cells of different tissues [60]. We currently lack a comprehensive catalog of the DNA binding sites for the two proteins as well as their chromatin infrastructure in neoplasia.

While there are known examples of functional redundancy and/or overlapping actions for KLF9 and KLF13, the current state of knowledge, specifically within the context of oncogenesis, suggests that there are functional differences and similarities between the two paralogs (reinforced by the results in Table 1 and summary in Figure 4). The GI tract, prostate, and glial cells manifest the anti-oncogenic effects of KLF9 and KLF13; the endometrium exhibits the anti-oncogenic effects of KLF9 and the pro-oncogenic effects of KLF13; whereas, both KLF9 and KLF13 are pro-oncogenic in oral squamous carcinoma. The basis for these differences is undoubtedly complex, especially considering that both genes/proteins are co-expressed in many cells and tissues, albeit with differing abundances. Some of the ways to get at this question include Chip-Seq, single cell RNA-seq, and single cell proteomics studies for multiple normal and tumor cell types and tissues simultaneously for both KLF9 and KLF13. Such approaches would also clarify why particular tumors display decreased or increased CpG methylation (Table 1).

Mass spectrometry has identified multiple sites of post-translational modification for KLF9 and KLF13 proteins; however, we know little about the functional relevance of these sites, especially with respect to neoplasia and responses to cancer treatments. A similar argument can be made for the interactomes of KLF9 and KLF13. It is striking how many different nuclear DNA-binding proteins physically interact with KLF9, while there are many more post-translational modifications of KLF13 than of KLF9. It is likely that KLF9, with its rigidly conserved linear protein sequence, interacts with different individual proteins across the entirety of its sequence. Like the example of KLF13 and the CCL5 promoter, KLF9 is likely to be a “lynchpin” for higher order protein complexes in chromatin. How these complexes work in cancer cells of any tissue is under-explored but of clear importance. It seems reasonable that such complexes, along with changes in KLF phosphorylation and acetylation status (more for KLF13 than KLF9), will underpin the convergence of signaling pathways as well as growth and nutrient signals at the level of KLF target genes in pre-cancerous and cancer cells. The ties between KLF9 and KLF13 proteins and oxidative stress and anti-oxidative stress are likely to involve similar signaling and co-factor convergence in chromatin. Further elucidation of the details of such pathways has the potential to enable new therapies to augment catastrophic oxidative stress and apoptosis of specific cancer cell types during treatment.

**Figure 5:**
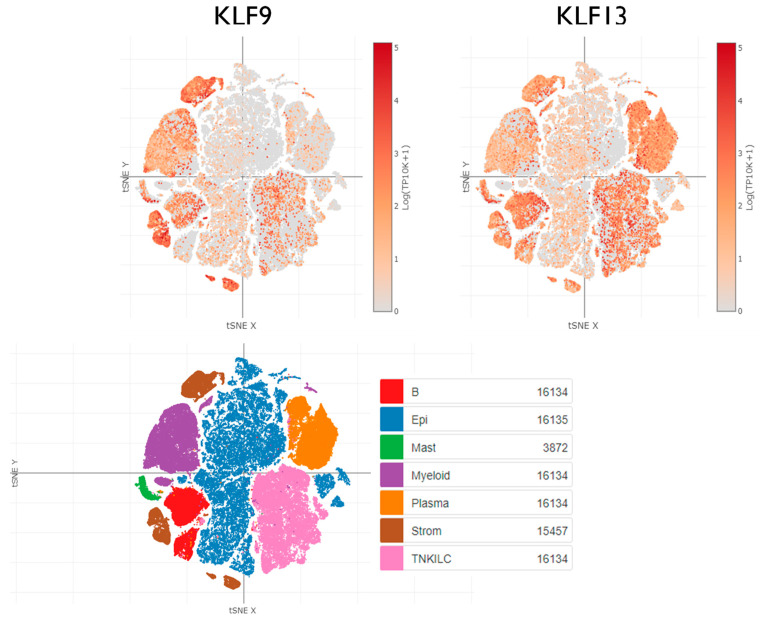
Cells expressing *KLF9* and *KLF13* transcripts in human colon tumors and associated normal colon tissue (Human Colon Cancer Atlas (scRNAseq) within the Single Cell Portal was queried) (https://singlecell.broadinstitute.org/single_cell, accessed on 16 May 2022) [232]. Data represent a global view of the sampled colon cells (t-distributed Stochastic Neighbor Embedding, tSNE). Key: B, B cells; Epi, epithelial cells; Mast, mast cells; Myeloid, myeloid cells; Plasma, plasma cells; Strom, all stromal cell types—fibroblasts, endothelial, pericytes, etc; TNKILC—T, NK, ILC cell types.

The extent of KLF9 and KLF13 co-expression and co-functionality in tumor cells, as well as in the non-malignant cells residing within the tumor microenvironment, is another key question to be addressed. To date, this information is absent for KLF9 and KLF13 in most human cancers. Perusal of the Human Colon Cancer Single Cell Atlas shows how important these types of analyses will be in forwarding this field. As evident in Figure 5, in human colon cancers, there is significant co-expression of *KLF9* and *KLF13* genes in the tumor (non-malignant) stroma, with the tumor epithelium relatively devoid of these transcripts (as expected for putative tumor suppressors). Further, the co-expression of *KLF9* and *KLF13* transcripts is evident in the tumor-associated immune cells (e.g., B cells, mast cells, myeloid cells, and T cells), whereas *KLF13* mRNA predominates in the tumor-associated plasma cells.

The expression of KLF9 and KLF13 in tumor-associated immune cells and tumor stroma, coupled with the known biology of these proteins in circulating immune cells, makes a strong case for focusing on the functions of these two KLFs in the tumor microenvironment of solid tumors and blood cancers. Perhaps these two KLFs will turn out to be important effectors of the immune-malignant cell network in tumors [232]. Clearly, we do not know enough about the specifics of KLF9 and KLF13 actions in the tumor immune cell microenvironment, a deficiency that should be rectified.

A corollary hypothesis is that the two KLFs may be useful biomarkers for predicting response to immune checkpoint blockade, depending upon the tumor type. In this regard, the work of Brown et al. [168], showing a suppressive effect of KLF9 on PD-L1 expression in the neoplastic mouse colon and in human colon cancer cell lines, supports this idea. Lastly, it is possible that KLF13 impacts tumoral CCL5 expression, with the latter molecule having major effects on immune cell infiltration into multiple tumor types, and with predicted positive as well as negative effects on tumor immune response [233,234].

Similar questions are clear for tumor-associated pre-adipocytes and adipocytes and their corresponding KLF9 and KLF13 expression and actions. The roles (similar or different) of KLF9 and KLF13 in cancer stem cells and whether reductions or absence of KLF expression drives tumor cell metastasis also requires follow up.

In the murine hippocampal cell line HT22, the *KLF16* gene promoter region is a target for repression of both KLF9 and KLF13 [123]. In these same cells, KLF9 is itself a repressor of the *KLF13* gene promoter [121]. This complex KLF circuitry has not been examined in any other biological context, such as in neoplasia. However, KLF16 has itself received much recent attention as a strong tumor promoting gene in multiple cancer types [235,236,237,238]. It is therefore tempting to speculate that the down regulation of KLF9 and/or KLF13 in pre-neoplastic cells leads, in turn, to synergistic inductions in KLF16 expression and consequent tumor promotion. Such a tumor inductive scenario (if confirmed) can be approached in two ways, i.e., targeting the suppressors of KLF9 and KLF13 and targeting KLF16.

## 5. Conclusions

Lastly, with respect to the future drugging of KLF9 and KLF13 for possible applications in cancer therapies, one goal would be to identify small molecule inducers of these mRNAs/proteins in pre-neoplastic and malignant cells. Thyroid hormone receptor (TRβ) agonists may prove particularly useful for KLF9. Epigenetic modifiers, such as HDAC inhibitors, may also work, depending on the malignancy under consideration. As described earlier, KLF9 appears to be regulated by numerous cognate miRs that target its mRNA. The miR matrix for KLF9 in lung cancers is particularly striking and large, and thus is a potential drug target in this malignancy, providing KLF9 is indeed confirmed to be beneficial to lung cancer patient prognosis. Anti-miRs with therapeutic efficacy and tumor-specificity could be designed to selectively enhance KLF9 protein translation in tumors, as, for example, in antibody-drug conjugates. Clofoctol, an antibiotic, is known to upregulate *KLF13* gene expression in glioma stem cells [156] and in mouse livers and kidneys [92]. Perhaps this drug or a suitable derivative may eventually provide a gateway to therapeutically induce KLF13 in neoplastic cells and/or tumor-associated immune cells. The increased interest that KLF9 and KLF13 have received in the oncology world portends an even more exciting future for these proteins in cancer prevention and treatment.

## Figures and Tables

**Table 1:** Methylation of *KLF9* and *KLF13* gene promoters in specific cancers ^a^.

*KLF9* ^b^	N ^c^	N ^c^	Cancer ^d^	Normal ^d^	*p* Value
Colon Cancer	309	38	0.0568	0.0504	<0.05
Esophageal Cancer	186	16	0.0545	0.0444	<0.05
Kidney Clear Cell Carcinoma	323	160	0.0557	0.0510	<0.05
Kidney Papillary Cell Carcinoma	276	45	0.0503	0.0449	<0.01
Hepatocellular Carcinoma	380	50	0.0557	0.0492	<0.01
Lung Squamous Cell Carcinoma	370	42	0.0569	0.0465	<0.01
Thyroid Cancer ^e^	515	56	0.0490	0.0548	<0.01
***KLF13* ^b^**	**N ^c^**	**N ^c^**	**Cancer ^d^**	**Normal ^d^**	***p* value**
Breast Cancer ^e^	794	96	0.1675	0.1752	<0.01
Cholangiocarcinoma ^e^	36	9	0.1466	0.1682	<0.05
Kidney Clear Cell	323	160	0.1422	0.1102	<0.01
Kidney Papillary	276	45	0.1313	0.1153	<0.01
Hepatocellular Carcinoma ^e^	380	50	0.1431	0.1666	<0.01
Pheochromocytoma & Paraganglioma ^e^	184	3	0.1236	0.1718	<0.01
Endometrial Cancer ^e^	436	46	0.1216	0.1711	<0.01

^a^ data were extracted from MethHC 2.0. (https://awi.cuhk.edu.cn/~MethHC/methhc_2020/php/index.php, accessed on 1 September 2023) [105,106]. ^b^ Cancers in which *KLF9* or *KLF13* gene promoter regions exhibited differential methylation compared to corresponding normal tissues. ^c^ Number of tumors or corresponding normal tissue samples examined. ^d^ Mean methylation level. ^e^ Tumors had less methylation of gene promoter region than did the corresponding normal tissue.
